# 影响肺癌手术住院费用和快速康复的临床因素分析

**DOI:** 10.3779/j.issn.1009-3419.2014.07.06

**Published:** 2014-07-20

**Authors:** 建华 苏, 鹏铭 喻, 渝斌 周, 强 蒲, 成奇 何, 伦旭 刘, 国卫 车

**Affiliations:** 1 610041 成都，四川大学华西医院康复科 Department of Rehabilitation Medicine, West China Hospital, Sichuan University, Chengdu 610041, China; 2 610041 成都，四川大学华西医院胸外科 Department of Thoracic Surgery, West China Hospital, Sichuan University, Chengdu 610041, China

**Keywords:** 住院费用, 快速康复, 外科治疗, 肺癌, In-hospital cost, Fast-track recovery, Surgery treatment, Lung neoplasms

## Abstract

**背景与目的:**

肺癌手术方式变化对其住院费用和快速康复的影响不太明确，本研究旨在探讨肺癌患者外科治疗住院费用和术后快速康复的临床影响因素。

**方法:**

收集2010年1月-2011年10月华西医院胸外科单个医疗组176例肺癌手术病例的临床资料进行分析。

**结果:**

胸腔镜肺叶切除术组住院费用（video-assisted thoracic surgery, VATS）（47, 308.21 ¥）高于开胸手术组（45, 664.31 ¥）（*P*=0.007）；体重指数（body mass index, BMI）≥24 kg/m^2^患者的住院费用（51, 186.99 ¥）高于BMI＜24 kg/m^2^（41, 701.64 ¥）的肺癌患者（*P*=0.032）。住院日VATS手术组（5.70 d）短于开胸手术组（7.10 d）（*P*＜0.001）。

**结论:**

手术方式和肺康复训练有助于肺癌手术患者术后的快速康复，但也增加了住院费用。

## Abstract (en)

**Background and objective:**

It is unknown that the postoperation fast-track recovery and in hospital cost of the lobectomy in lung cancer, we explored the influencing factor of postoperative fast-track recovery and in hospital cost after undergoing lobectomy for lung cancer.

**Methods:**

We retrospectively reviewed the medical records of all patients (*n*=176) who underwent lobectomy for lung cancer between January 2010 and November 2011 by a thoracic surgeon.

**Results:**

The hospital costs of video-assisted thoracic surgery (VATS) lobectomy (47, 308.21 ¥) is significantly higher than open lobectomy (45, 664.31 ¥)(*P*=0.007). The hospital costs of body mass index (BMI) ≥ 24 kg/m^2^ (51, 186.99 ¥) is significantly higher than BMI < 24 kg/m^2^ (41, 701.64 ¥)(*P*=0.032). The hospital stay of VATS lobectomy (5.70 d) is significantly less than open lobectomy (7.10 d)(*P* < 0.001).

**Conclusion:**

These findings indicate that preoperative pulmonary rehabilitation and VATS lobectomy is contributed to fast-track recovery for patients who undergo lobectomy, but increase the hospital costs.

肺癌的治疗仍是以手术为主的多学科综合治疗。外科治疗费用和术后并发症是肺癌患者关心的主要问题，也是医患产生矛盾的关键所在。分析当前外科治疗肺癌的住院费用构成和影响术后的并发症成为必然。肺癌患者术前的嗜好（如吸烟^[1]^）和身体状况（如是否肥胖^[2]^）及手术方式和肺康复训练^[3, 4]^对住院费用和术后快速康复的确切影响仍不清楚。有研究^[5]^表明肺癌胸腔镜肺叶切除术（video-assisted thoracic surgery, VATS）增加了手术材料费，降低了药费，而总体费用却变化不大，甚至还有降低。但是国内却没有相关报道。本研究回顾性分析了肺癌患者临床特征、肺功能状态及肺康复训练、手术方式及术后并发症等对住院费用和快速康复的影响。

## 1 资料与方法

### 1.1 临床资料和分组

纳入标准：①病理学检查为原发性肺癌患者；②术前检查无手术绝对禁忌症并签署肺康复训练同意书；③VATS或开胸手术+系统淋巴结清扫术。排除标准：①需要行全肺切除和需要行肺动脉和支气管袖式成形肺叶切除术；②术后需要再次止血；③术中出血量超过1, 000 mL和VATS手术中转开胸；④不愿意接受肺康复训练。

连续纳入2010年1月-2011年10月四川大学华西医院胸外科单个医疗组176例肺癌患者，其中男性101例，女性75例，平均年龄（56.85±9.31）岁；有吸烟史的患者97例，其中≥400年支的有64例；VATS手术73例，开胸手术103例；鳞癌63例，腺癌107例，腺鳞癌5例，小细胞癌1例；Ⅰ（Ⅰa+Ⅰb）期65例，Ⅱ（Ⅱa+Ⅱb）期49例，Ⅲ（Ⅲa+Ⅲb）期60例，Ⅳ期2例。根据“四川大学华西医院康复医学中心-术前心肺功能康复评定报告”进行心肺运动试验评估心肺功能并收集病例的全部临床资料。对术前吸烟≥400年支且戒烟时间小于2周的64例患者进行3 d-7 d的肺康复训练。

### 1.2 方法

#### 1.2.1 基础肺功能（pulmonary function test, PFT）检测指标

由四川大学华西医院肺功能室完成并出具实验报告；肺通气功能（肺容积、肺通气量、小气道功能、呼吸动力学、吸入气体分布、呼吸肌功能）和肺换气功能（弥散功能、通气血流比值）进行测定和评估。

#### 1.2.2 心肺运动试验方法检测及项目

①器械型号及厂家：运动仪器endorphin，检测仪器konica minolta pulsox-300；②检测方法：阻力均为27瓦，要求6 min之内尽可能快速的运动，如果患者觉得累或气紧可以减慢速度或者停止运动，恢复后继续运动；③检测项目：从患者静息开始检测心律和血氧饱和度到运动结束停止检测，Borg呼吸困难评分，患者6 min钟运动距离（6 min walk distance, 6-MVD）和能量消耗。

#### 1.2.3 肺康复方法

##### 1.2.3.1 药物康复治疗

①抗感染治疗：头孢美唑钠注射液2 g，每天两次，静脉滴注；②雾化吸入支气管扩张剂和糖皮质激素治疗：爱全乐+普米克令舒各2 mL，每天2次，雾化吸入；③祛痰治疗：盐酸氨溴索注射液60 mg，每天2次，静滴；④静脉应用糖皮质激素治疗：甲基强的松龙40 mg，每天2次，静滴。

##### 1.2.3.2 肺康复物理训练^[6]^

呼吸生理适应训练：①腹式呼吸训练：患者取平卧位，集中精神，全身放松，经鼻缓慢深吸气到最大肺容量后稍屏气，然后用口缓慢呼气，吸气时膈肌下降，腹部外凸；呼气时膈肌上升，腹部内凹。连续进行20次-30次（总时间约15 min-30 min），每天早、晚各进行1次训练。②吸气训练器训练（VOLDYNE5000呼吸训练器）：患者取坐位，正常呼气后用嘴含紧吸气嘴，以大的吸气量把小球吸上筒腔的顶端不动，屏气2 s-3 s，然后移开吸气嘴，缩唇慢呼气，重复练习，每天4次，每次20 min。下肢耐力训练：①功率自行车运动训练：患者自行调控速度，在承受范围内逐步加快步行速度及自行车功率。运动量控制在BORG评分5分-7分之间，若在运动过程中有明显气促、腿疲倦、血氧饱和度下降（＜88%）或其它并存疾病引起身体不适，告诉患者休息，待恢复原状后再继续进行训练。每次约15 min-20 min，2次/天，疗程为2周。②爬楼梯训练：在专业治疗师陪同下进行，在运动过程中调整呼吸节奏，采用缩唇呼吸，用力时呼气，避免闭气，稍感气短时可坚持进行，若有明显呼吸困难，可做短暂休息，尽快继续运动。每次约15 min-30 min，2次/天，疗程为2周。

##### 1.2.3.3 肺康复方案^[7]^

戒烟不足2周或吸烟指数≥400年支的患者行抗感染治疗、雾化吸入支气管扩张剂和糖皮质激素治疗、祛痰治疗、腹式呼吸训练、下肢耐力训练方案治疗的同时，于术前1 d进行静脉应用糖皮质激素治疗。

#### 1.2.4 手术方法

VATS组应用单向式胸腔镜肺叶切除法+系统淋巴结清扫^[8]^。开胸组应用常规后外侧切口，肺叶切除术+系统淋巴结清扫。系统淋巴结清扫左侧必须清扫第5、6、7、8、9、10组淋巴结，右侧包括第2、3、4、7、8、9、10组淋巴结。

#### 1.2.5 术后处理方法^[7]^

术后疼痛处理均应用镇痛泵（盐酸曲马多注射液，1 mg/h-1.5 mg/h），均早期促使患者下床活动。必要时应用非甾体类止痛药（氨酚羟考酮片或布洛芬缓释胶囊）。镇痛泵于胸腔引流管拔除的同时也一起停止。两组患者均根据胸腔引流气体和液体量决定行胸部X线片检查时间，并根据胸部X线片结果决定是否拔除胸腔引流管。

#### 1.2.6 观测指标

##### 1.2.6.1 术后并发症^[7]^

包括（1）腹泻；（2）过敏反应；（3）皮下气肿；（4）心律失常；（5）小便失禁；（6）术后胸腔积气：胸部X线片提示：胸腔积气＞30%；（7）术后胸腔积液：胸部X线片提示：胸腔积液中量以上；（8）肺部感染^[8]^：①明确的病原学证据；②影像学提示肺不张或大片状影；③发热；④白细胞总数大于10, 000/mL或15, 000/mL。

##### 1.2.6.2 住院日

住院日=术前等待时间+术后住院时间。

##### 1.2.6.3 住院总费用

包括：检查费、手术费、西药费、材料费（手术中所用切割缝合器）、床位费等。肺康复费用包括：心肺运动试验检测费用、物理治疗费用、呼吸训练器及训练费用、药费。

### 1.3 统计学分析

应用SPSS 13.0统计软件分析数据，均数组间比较采用*t*检验，非正态分布的计量数据比较采用秩和检验，率的比较采用卡方检验，*P*＜0.05为差异有统计学意义。

## 2 结果

### 2.1 176例肺癌患者临床特征基本描述

见表 1。住院天数和住院费用不符合正态分布，选择中位数来描述，则住院天数（d）的中位数为6.00，住院费用（¥）的中位数为40, 692.00，肺癌患者术后72 h内白细胞均数为（14.94±5.03）×10^9^/L（95%CI: 14.51-16.07）。

**1:** 176例肺癌患者临床特征基本描述 The characteristics of 176 cases in patients with lung cancer

Characteristic	Mean	SD	95%CI
Age (yr）	56.85	9.31	55.46-58.24
BMI (kg/m^2^)	22.86	2.94	22.42-23.30
PEF (L/min)	371.93	111.77	355.30-388.56
WBC (×10^9^/L)	14.94	5.03	14.51-16.07
MMEF (%)	57.57	26.43	53.20-61.94
V50 (%)	64.52	31.33	59.34-69.70
V25 (%)	49.25	27.56	44.69-53.80
DLCO (%)	104.28	19.22	101.10-107.10
6-min walk distance (m)	639.38	87.99	626.26-652.51
FEV1 (%)	95.03	16.87	92.26-97.80
BMI: body mass index; PEF: peak expiratory flow; MMEF: maximal midexpiratory flow; FEV1: Forced expiratory volume in one second; WBC: White blood cell.			

### 2.2 影响住院费用因素分析

体重指数（body mass index, BMI）≥24 kg/m^2^（41, 701.64 ¥）组的住院费用高于BMI＜24 kg/m^2^（51, 186.99 ¥）的肺癌患者（*P*=0.032）；有并发症患者（47, 623.32 ¥）的住院费用高于无并发症的肺癌患者（42, 809.07 ¥），但差异没有统计学意义（*P*=0.517）。

### 2.3 住院期间转科手术方式对住院费的影响

若只将胸外科出院时住院费用以手术方式进行比较，则住院费在开胸组（45, 664.31 ¥）高于VATS组（44, 131.59 ¥）（*P*＜0.001）；若住院背景相同（转科与未转科分别分析），则住院费用在VATS是开胸费用1.086, 4倍（47, 308.21 ¥ *vs* 45, 664.31 ¥, *P*=0.007）。若手术方式相同，住院费用在转科比未转科的对数值高0.084，即转科的住院费用是未转科的1.213倍（*P*＜0.001）。

### 2.4 住院日影响因素分析

VATS手术组的住院日（5.70 d）短于开胸手术（7.10 d）（*P*＜0.001）。FEV1≥80%住院日（6.13 d）短于FEV1＜80%（7.09 d），但无统计学差异（*P*=0.095）。而吸烟指数多少（*P*=0.316）和并发症有无（*P*=0.341）并没有影响本组病例的住院日。

### 2.5 影响并发症的相关因素分析

若将WBC≥10, 000/mL作为标准则并发症有80例，若WBC≥15, 000/mL作为标准则仅有19例。不论白细胞计数是WBC≥10, 000/L或WBC≥15, 000/mL，仅有吸烟指数与并发症有关，且是高吸烟指数（≥400年支）的并发症低于低吸烟指数（＜400年支）（*P*=0.017, *P*=0.018）（表 2）。这个结果是因为吸烟指数≥400年支均进行了肺康复训练，可见肺康复训练有助于降低吸烟带来的并发症。

**2:** 影响术后并发症的临床因素 The impact factors of postoperation complications in lung cancer

Factor	Complications	WBC≥10, 000/mL				WBC≥15, 000/mL		
		Mean	*t*	*P*		Mean	*t*	*P*
6-min walk distance (m)	No	644.44	0.828	0.409		643.03	1.576	0.117
	Yes	633.38				609.47		
BMI (kg/m^2^)	No	22.75	-0.535	0.594		22.91	0.645	0.52
	Yes	22.99				22.45		
PEF (L/min)	No	368.18	-0.487	0.627		372.8	0.296	0.767
	Yes	376.44				364.74		
Smoking index	No	847.29	2.435	0.017		784.3	2.398	0.018
	Yes	626.41				455.69		

## 3 讨论

近20年来，医疗设备更新和技术的进步（以腔镜技术和器械为代表），极大地促进肺癌外科治疗的发展。微创技术的应用不但促进快速康复，也使医疗成本增高。理论上新技术必然推进理念的更新，现实是全胸腔镜肺叶切除术已在临床广泛应用，而术前准备和术后处理仍使用开胸的理念和方法，这必然使新技术的优势不能充分发挥和评估。研究与腔镜技术相适应的系统处理措施，需要对影响肺癌外科治疗的住院费用和快速康复的因素进行分析。国内关于此项的研究尚没有报道，国外也只是研究新的药物或术式对医疗费用的影响^[5]^。因此，我们应用华西医院数据库的优势，对华西医院胸外科住院病例肺癌治疗的费用及其影响因素进行了客观分析。

研究^[9]^发现吸烟指数高和戒烟时间短与肺癌术后并发症的发生密切相关，吸烟指数高的患者并发症低，且于并发症中白细胞计数（≥15, 000/mL或≥10, 000/mL）标准无关，这个结果是因为我们在临床工作中对吸烟指数≥400年支的患者，术前进行了肺康复训练（3 d-7 d），提示肺康复训练有助于降低高危人群的肺部并发症。研究^[10]^表明术前肺康复训练可以使肺癌患者术后肺功能降低的程度减少，促进快速康复表现在缩短住院时间。

176例肺癌患者术后72 h内白细胞计数均在15, 000/L左右，提示将WBC≥10, 000/mL作为外科术后并发症或肺部感染的标准是不恰当的，我们的研究发现不同的标准同一组病例并发症发生率（45.46%, 80/176; WBC≥10, 000/mL）下降到10.80%（19/176; WBC≥15, 000/mL），发生例数也减少4/5，可见将WBC≥15, 000/mL作为外科术后并发症的诊断标准比较恰当。问题是本研究中有VATS手术73例，白细胞（14, 005/mL）小于开胸手术（15, 328/mL），关于WBC≥15, 000/mL是否对不同手术方式选择标准，需要进一步研究。

研究^[11]^发现吸烟指数可以增加肺癌术后的并发症，而并发症的发生必然导致住院日的延长，而我们的研究发现只有手术方式与住院日有关，VATS术后住院日短于开胸手术，这与大多数结果^[12, 13]^一致。而并发症没有增加住院日，应该和高危因素患者术前进行肺康复训练有关^[14, 15]^，一方面降低了并发症的发生率，另一方面也降低了并发症的严重程度。

我们的总体研究结果中有开胸的手术费用高于VATS的住院费用，分析可能与两种因素有关，一是转科病例中多数在开胸组，而这部分病例均病情复杂，内科住院期间药费高和重复检查增加了住院费用；二是开胸病例有时也用腔镜下的切割缝合器使手术材料费用和VATS手术差别减小。分别排除住院期间转科因素的影响，仍发现VATS手术费用是开胸手术的1.1倍；而转科病例住院费用则是正常入院手术的1.213倍，提醒我们合理的收治病例，降低非必须医疗费用的空间是有的。另外，患者的BMI≥24 kg/m^2^也增加了住院费用，事实上BMI并没有增加术后的并发症^[16]^，可能于住院过程中药费和手术材料费增加有关。

总之，回顾性分析研究尽管不能提供肯定的结论，但从临床角度反映了一些问题。值得肯定的是肺癌患者高危人群的术前肺康复训练是降低并发症；从病例住院费用上看，可以从不同的角度降低费用，使肺癌的治疗方案具有更好的社会效益和卫生经济效益。
